# 马立巴韦治疗异基因造血干细胞移植后难治与药物不耐受CMV血症和CMV病25例临床分析

**DOI:** 10.3760/cma.j.cn121090-20240919-00355

**Published:** 2024-11

**Authors:** 薇 马, 志杰 魏, 岳 卢, 建平 张, 瑞娟 孙, 敏 熊, 葭蕤 周, 磊 董, 松 薛, 星玉 曹

**Affiliations:** 1 河北燕达陆道培医院造血干细胞移植科，廊坊 065201 Department of Bone Marrow Transplantation, Hebei Yanda Lu Daopei Hospital, Langfang 065201, China; 2 北京陆道培医院移植科，北京 100176 Department of Bone Marrow Transplantation, Beijing Lu Daopei Hospital, Beijing 101102, China

**Keywords:** 巨细胞病毒, 异基因造血干细胞移植, 马立巴韦, Cytomegalovirus, Allogeneic hematopoietic stem cell transplantation, Maribavir

## Abstract

**目的:**

探讨马立巴韦治疗异基因造血干细胞移植（allo-HSCT）后难治性、对常规抗病毒药物不耐受的巨细胞病毒（cytomegalovirus，CMV）血症和CMV病的安全性和有效性。

**方法:**

回顾性分析2024年4月至2024年9月在河北燕达陆道培医院马立巴韦治疗allo-HSCT后的难治性、对常规抗病毒药物不耐受的CMV血症和CMV病的结局。

**结果:**

接受马立巴韦治疗的25例患者，其中亲缘单倍型移植21例，同胞全合移植2例，非血缘移植2例。1次移植21例，2次移植2例和3次移植2例。患者发生CMV血症的中位时间为移植后120.5（6～298）d，血浆CMV最高拷贝中位数为6 400（1 100～650 000）拷贝/ml。6例患者诊断CMV病。患者在CMV感染后的9.5（1～41）d开始使用马立巴韦。使用马立巴韦持续中位时间为11.5（6～43）d。25例患者治疗均有效。2例患者发生1级的味觉异常，1例发生2级骨髓抑制。

**结论:**

allo-HSCT后难治性、药物不耐受的CMV血症和CMV病患者应用马立巴韦治疗安全有效。

## Abstract (en)

**Objective:**

To investigate the safety and efficacy of maribavir for the treatment of CMV viremia and CMV disease refractory or intolerant to conventional antiviral drugs after allogeneic hematopoietic stem cell transplantation（allo-HSCT）.

**Methods:**

This study retrospectively analyzed the clinical characteristics and outcomes of CMV viremia and CMV disease refractory or intolerant to conventional antiviral drugs after allo-HSCT treated with maribavir at Hebei Yanda Lu Daopei Hospital from April 2024 to September 2024.

**Results:**

A total of 25 patients received maribavir, including 21 haploidentical transplants, two sibling HLA-matched transplants, and 2 HLA-matched unrelated transplants. Among them, 21, 2, and 2 patients received the first, second, and third transplants, respectively. The median time to the onset of CMV viremia and CMV disease was 120.5（6–298）days post-transplantation. The median peak plasma CMV copy number was 6 400 copies/ml（range: 1 100–650 000 copies/ml）. Six patients were diagnosed with CMV disease. Maribavir was administered after a median of 9.5（1–41）days after CMV infection. The median duration of maribavir administration was 11.5（6–43）days. Post-treatment, maribavir was effective in 25（100％）patients. Two patients experienced grade 1 taste abnormalities, and one patient experienced grade 2 myelosuppression.

**Conclusion:**

The application of maribavir after allo-HSCT for treating refractory, drug-intolerant CMV viremia and CMV disease is safe and effective.

巨细胞病毒（CMV）感染是异基因造血干细胞移植（allo-HSCT）后常见的影响预后、甚至危及生命的并发症[1]。CMV感染增加移植后非复发相关死亡率（NRM）、移植物抗宿主病（GVHD）发生率和继发的细菌与真菌感染发生率[2]–[5]。Marty等[6]的研究显示，使用来特莫韦进行CMV预防可以显著降低移植后24周CMV感染的发生率，从安慰剂组的60.6％降至37.5％；然而，停药后来特莫韦组的迟发CMV感染率上升至18.9％。另一方面更昔洛韦和膦甲酸钠等常规抗CMV药物有骨髓抑制和肾毒性，且常规抗CMV药物对于难治性CMV感染/CMV病疗效有限[7]–[8]。马立巴韦是一种新型的抗CMV病毒药物，可以直接作用于CMV蛋白酶UL97，影响病毒DNA复制、DNA包壳和核出口，具有多模式的抗CMV活性[9]–[10]，与更昔洛韦和膦甲酸钠无交叉耐药。马立巴韦在中国已于2023年12月份上市，但是目前尚缺乏在国内临床应用的数据。本研究回顾性分析了25例应用马立巴韦治疗的allo-HSCT后CMV感染患者的临床资料，探讨移植后应用马立巴韦治疗难治性、对常规抗病毒药物不耐受的CMV感染的安全性和有效性。

## 病例与方法

1. 病例资料：回顾性纳入2024年4月至2024年9月在河北燕达陆道培医院allo-HSCT后难治性、对常规抗病毒药物不耐受的CMV血症和CMV病且接受马立巴韦治疗的25例患者，分析患者临床特征、用药情况和相关实验室指标，评估马立巴韦的安全性和有效性。

2. 移植方案：采用以全身放疗（TBI）、白消安（Bu）或美法仑（Mel）为基础的预处理方案。25例患者均联合抗胸腺/淋巴细胞球蛋白（ATG/ALG）。患者接受G-CSF动员的骨髓+外周血干细胞或G-CSF动员的外周血干细胞，亲缘单倍型移植患者在+1 d予配型3/6～6/6相合的脐带血或另一亲缘半相合供者骨髓作为第三方细胞辅助回输。

3. GVHD的预防：采用环孢素（CsA）/他克莫司（FK506）、吗替麦考酚酯（MMF）及短程甲氨蝶呤（MTX）方案预防GVHD。从−9 d开始予CsA或FK506，初始剂量分别为1.25 mg/kg每12 h 1次静脉滴注、0.015 mg/kg静脉维持24 h，根据药物浓度调节剂量。MTX 15 mg/m^2^，+1 d；10 mg·m^−2^·d^−1^，+3 d、+6 d、+11 d。

4. CMV感染的预防、监测和治疗：−9 d～−2 d给予更昔洛韦5 mg/kg静脉滴注每日2次。+1 d～+100 d给予口服来特莫韦预防，≥12岁儿童和成人剂量为480 mg/d，如与CsA合用，剂量调整为240 mg/d；6个月～<12岁儿童根据体重调整剂量。23例患者接受来特莫韦预防CMV，2例患者因个人原因未采用来特莫韦预防。

供患者在移植前均采用ELISA法检测血清抗CMV-IgG和IgM抗体。从中性粒细胞植入至+100 d，通过实时荧光定量PCR的方法每周2次检测患者血浆CMV-DNA拷贝数，检测灵敏度为1×10^3^拷贝数/ml；+100 d后根据患者免疫功能和免疫抑制剂使用情况确定监测频率。

患者确诊CMV感染后，常规治疗方案包括：静脉滴注更昔洛5 mg/kg，每12 h 1次；膦甲酸钠60 mg/kg，每8 h 1次；静脉输注人免疫球蛋白0.4 g/kg每日1次，4～5 d；CMV丙种球蛋白150 mg/kg隔日1次。当出现以下情况之一时应用马立巴韦：①诊断难治性CMV感染；②常规治疗不耐受：例如骨髓抑制，肾功能损害等。马立巴韦的剂量如下：≥12岁儿童和成人为400 mg每日2次口服；<12岁的儿童剂量参考https://clinicaltrials.gov/ct2/show/NCT05319353[11]。

5. 其他相关指标的监测和感染的预防：淋巴细胞亚群的测定采用流式细胞术进行分析。患者从中性粒细胞植入开始通过实时荧光定量PCR监测血浆EBV-DNA拷贝数。+1 d开始口服阿昔洛韦预防单纯疱疹和水痘带状疱疹。常规用复方磺胺甲噁唑预防肺孢子菌肺炎。

6. 相关定义：粒细胞植活定义：连续3 d中性粒细胞计数≥0.5×10^9^/L的第1天为粒细胞植活时间。血小板植活：无血小板输注情况下，血小板计数连续7 d≥20×10^9^/L的第1天为血小板植活时间。CMV感染包括CMV血症和CMV病，相关定义见参考文献[12]。难治性CMV感染：接受合理的抗CMV治疗2周后，CMV病毒载量维持不变或上升。难治性CMV病：接受合理的抗CMV治疗2周后，CMV病的症状、体征无改善或持续进展[13]。急性移植物抗宿主病（aGVHD）按照Glucksberg分度。慢性移植物抗宿主病（cGVHD）按照NIH标准进行分度[14]。移植相关血栓性微血管病（TA-TMA）诊断标准见2023年国际共识[15]。肝窦阻塞综合征（SOS）诊断按照2023年EBMT的诊断标准[16]。无白血病生存（LFS）时间定义为造血干细胞回输结束至死亡、复发或者末次随访时间。总生存（OS）时间定义为造血干细胞回输结束至末次随访或死亡的时间。复发：缓解后再次出现骨髓中白血病细胞>5％或髓外白血病。非复发死亡：因非复发或非疾病进展原因造成的死亡。不良反应按照CTCAE5.0版进行分级。

7. 随访及观察：采用查阅住院、门诊病历和电话询问的方式进行随访。中位随访时间为移植后180（26～411）d。随访时间截至2024年10月15日。

## 结果

1. 患者基本资料：25例患者中，男14例，女11例，中位年龄31（3～61）岁。患者基本临床特征见表1。患者原发病以急性髓系白血病（AML）为主（56％，14/25），移植类型主要为亲缘单倍型移植（88％，22/25）。84％的患者为第1次移植。预处理方案以Bu为主的15例（60％），以TBI为主的9例（36％）。回输单个核细胞（MNC）中位数为9.7（4.6～26.5）×10^8^/kg；CD34^+^细胞中位数为6.79（2.9～19.04）×10^6^/kg；CD3^+^细胞中位数为2.17（0.56～5.31）×10^8^/kg。粒细胞植活中位时间为14.5（11～25）d；1例患者血小板未植活，其余24例患者血小板植活中位时间为15.5（9～126）d。

2. CMV感染情况和马立巴韦的治疗：移植前供受者CMV-IgG和IgM情况见表1。其中供者血清学阴性5例。23例患者接受来特莫韦预防CMV。25例患者移植后发生CMV感染的中位时间为120.5（6～298）d。血浆CMV最高拷贝中位数为6 400（1 100～650 000）拷贝数/ml。6例（24％）CMV病患者中，5例为CMV肺炎，1例为CMV肠炎。13例患者检测了UL97基因突变，结果均为阴性。

**表1:** allo-HSCT后难治性、对常规抗病毒药物不耐受的CMV血症和CMV病并接受马立巴韦治疗25例患者临床一般资料

临床特征	例数（％）
性别	
男	14（56.0）
女	11（44.0）
疾病诊断	
sAA	3（12.0）
ALL	5（20.0）
AML	14（56.0）
MDS	2（8.0）
CTCL	1（4.0）
HCT-CI评分	
0分	10（40.0）
1分	10（40.0）
2分	5（20.0）
移植类型	
亲缘单倍体	21（84.0）
非血缘	2（8.0）
同胞全合	2（8.0）
供受者CMV IgG关系
阴供阴	1（4.0）
阴供阳	3（12.0）
阳供阳	21（84.0）
移植次数	
1次移植	21（84.0）
2次移植	2（8.0）
3次移植	2（8.0）
预处理方案
以Bu为基础	15（60.0）
以TBI为基础	9（36.0）
以Mel为基础	1（4.0）
移植物类型	
骨髓+外周血	18（72.0）
外周血	7（28.0）
ATG种类	
抗人T细胞兔免疫球蛋白	11（44.0）
抗人T细胞猪免疫球蛋白	2（8.0）
兔抗人胸腺免疫球蛋白	12（48.0）
来特莫韦预防
有	23（92.0）
无	2（8.0）
马立巴韦合并用药
有合并用药	18（72.0）
无合并用药	7（28.0）

**注** allo-HSCT：异基因造血干细胞移植；CMV：巨细胞病毒；sAA：重型再生障碍性贫血；MDS：骨髓增生异常综合征；ALL：急性淋巴细胞白血病；AML：急性髓系白血病；CTCL：原发性皮肤T细胞淋巴瘤；HCT-CI：造血干细胞移植合并症指数；Bu：白消安；TBI：全身放疗；Mel：美法仑

25例患者中23例在接受马立巴韦治疗前均接受过传统抗CMV药物来特莫韦的治疗，其中3例出现肾脏损伤，4例出现全血细胞减少。接受马立巴韦治疗原因分为难治性CMV感染、传统抗病毒药物不耐受。马立巴韦应用距CMV感染诊断中位间隔9.5（1～41）d。马立巴韦中位治疗时间为11.5（6～43）d。马立巴韦开始治疗后7（3～22）d CMV-DNA拷贝数<1 000拷贝数/ml，25例患者应用马立巴韦后CMV-DNA均转为阴性。马立巴韦停药后1例在4 d后CMV-DNA拷贝数达到24 000拷贝数/ml，再次马立巴韦治疗13 d后CMV-DNA持续阴性。马立巴韦合并用药包括CMV特异性免疫球蛋白、免疫球蛋白、IL-2、膦甲酸钠。

1例CMV肠炎患者，临床表现为发热、腹痛和腹泻，每日大便量700～800 g，黄绿色浠水便。5例CMV肺炎患者中，表现为轻度胸闷和憋气、发热、咳嗽、咯痰、血氧饱和度下降（91％～94％）；25例患者中2例出现味觉异常，均为1级；1例发生2级骨髓抑制，马立巴韦停药14 d后血常规逐渐恢复正常。未出现其他不良反应。

3. 移植后发生CMV感染时免疫细胞亚群的监测：发生CMV感染时CD4^+^细胞中位数为38（1～99）个/µl，CD8^+^细胞87（15～690）个/µl。待CMV持续转阴后，CD4^+^、CD8^+^细胞数逐渐上升，CD4^+^细胞中位数为130（6～520）个/µl，CD8^+^细胞中位数为298（133～1315）个/µl。1例患者CD8^+^细胞监测持续低，停用马立巴韦4 d后再次血浆CMV复阳。

4. 移植后其他相关合并症情况：25例患者+100 d Ⅱ～Ⅳ度aGVHD累积发生率为25.0％（95％*CI* 11.7％～53.6％），Ⅲ～Ⅳ度aGVHD发生率为8.0％（95％*CI* 2.1％～30.0％）。移植后6个月cGVHD累积发生率为27.4％（95％*CI* 13.9％～54.1％），其中中重度cGVHD累积发生率为18.6％（95％*CI* 7.7％～45.0％）。+100 d EBV血症的累积发生率为25.0％（95％*CI* 11.7％～53.6％）。1例患者发生TA-TMA，1例发生侵袭性毛霉菌病，2例发生出血性膀胱炎，1例发生SOS，1例发生移植后淋巴细胞增殖性疾病，1例发生移植物功能不良。

5. 转归：共4例死亡，死亡原因分别为TA-TMA 1例，感染性休克1例，病毒性肺炎1例，Ⅳ度aGVHD 1例。25例患者中1例+60 d复发并生存，20例无病生存。6个月OS和LFS率分别为85.9％（95％*CI* 71.0％～100％）、81.5％（95％*CI* 65.0％～98.0％）。

## 讨论

CMV感染是allo-HSCT后常见的并发症[12]，在来特莫韦应用前时代，同胞全合、非血缘相合、含ATG预处理方案的亲缘单倍型和脐带血移植CMV感染发生率分别为18％～55％、65％～70％、60％～83％和70％～85％[17]–[21]。CMV病的发生率为10％～40％，最主要的类型是CMV肺炎，病死率高达70％[12]。而采用来特莫韦预防后，CMV的发生率明显降低。但在allo-HSCT后仍有39％的患者可以发生难治性CMV感染[12],[23]。难治性CMV感染中14.5％的患者是由于CMV病毒UL97和UL54基因突变造成的耐药[23]。由于我国广泛采用单倍型移植，难治性CMV感染发生率高于国外[23]。难治性CMV感染是非复发死亡的独立危险因素[20],[24]–[27]。

马立巴韦直接作用于CMV蛋白酶UL97，影响病毒DNA复制、DNA包壳和核出口，具有多模式抗CMV活性。在一项Ⅱ期前瞻性随机对照临床试验中，马立巴韦和缬更昔洛韦一线抢先治疗6周，治疗反应率分别为75％和48％，马立巴韦组有更多的胃肠道不良反应，而缬更昔洛韦组有更多患者发生骨髓抑制[28]。当用于难治CMV血症或者CMV病的治疗时，马立巴韦组和传统药物治疗组8周时CMV血症的清除率分别为55.7％和23.9％（*P*<0.001），且马立巴韦的肾毒性低于膦甲酸钠，分别为8.5％和21.3％；马立巴韦的中性粒细胞减少发生率低于缬更昔洛韦/更昔洛韦，分别为9.4％和33.9％[29]。对于CMV病患者，马立巴韦治疗后，89％患者症状可以得到改善或完全缓解，马立巴韦对CMV肺炎、CMV肠炎有效，即使在肠道GVHD导致吸收障碍的患者中，也仍具有良好的吸收效果，对CMV视网膜炎部分有效，但是对CMV脑炎无效[30]–[31]。

在本研究中23例患者为采用来特莫韦预防后突破性CMV感染。尽管其中13例UL97突变检测均为阴性，不排除存在UL54的突变或者免疫功能严重受损有关。因为UL54突变的检测在我中心尚未开展，因此无法证实。25例患者预处理均采用了ATG，2例为2次移植，2例为3次移植，可能会出现免疫功能的严重受损。

马立巴韦治疗后25例患者全部有效。其中3例患者血浆CMV拷贝数最高达到了400 000、630 000和650 000拷贝数/ml。CMV<1 000拷贝数/ml中位时间7（3～22）d，未出现肾脏损伤，1例患者出现骨髓抑制，停马立巴韦14 d后血常规恢复正常。2例患者出现味觉障碍，均为1级。马立巴韦显示了起效快、疗效好和安全的特点。停药后仅有1例患者出现了CMV病毒拷贝数的快速升高，但是再次给予马立巴韦后又快速转阴。25例患者马立巴韦中位治疗时间为11.5（6～43）d，远低于文献报道的中位时间75（5～177）d[30]。

在来特莫韦预防的患者中CMV特异性的细胞免疫重建延迟[32]。来特莫韦停药后CMV的晚期激活和体内CMV特异性的T细胞和记忆样NK细胞数量低和延迟恢复有关[33]。本研究中，25例患者CMV感染后CD4^+^和CD8^+^ T细胞水平均较低，1例患者因CD8^+^ T细胞重建延迟，CMV转阴后再次复发。CMV-DNA转阴的患者CD8^+^细胞呈恢复趋势，因此可以尝试对于CMV-DNA阴性且淋巴细胞恢复的患者尽早停药。

本研究存在一定的局限性。首先，作为单中心回顾性分析，样本量较少，随访时间较短，结论有待多中心、大样本、长期随访研究验证。其次，25例患者中仅有7例为马立巴韦的单药治疗，其他18例均有合并用药情况，马立巴韦单药疗效的评估存在偏倚。

总之，本中心的研究结果表明，allo-HSCT后无论难治、药物不耐受的CMV血症或CMV病，口服马立巴韦进行治疗均显示了良好的治疗效果，具有清除率高、中位清除时间短和不良反应小的特点。
